# Calibrating the zenith of dinosaur diversity in the Campanian of the Western Interior Basin by CA-ID-TIMS U–Pb geochronology

**DOI:** 10.1038/s41598-022-19896-w

**Published:** 2022-09-26

**Authors:** Jahandar Ramezani, Tegan L. Beveridge, Raymond R. Rogers, David A. Eberth, Eric M. Roberts

**Affiliations:** 1 Department of Earth, Atmospheric and Planetary Sciences, Massachusetts Institute of Technology, Cambridge, MA 02139 USA; 2 Department of Earth and Environmental Sciences, James Cook University, Townsville, QLD 4811 Australia; 3 Geology Department, Macalester College, Saint Paul, MN 55105 USA; 4 Royal Tyrrell Museum of Palaeontology, Drumheller, AB T0J 0Y0 Canada

**Keywords:** Stratigraphy, Sedimentology, Geochemistry, Palaeoecology

## Abstract

The spectacular fossil fauna and flora preserved in the Upper Cretaceous terrestrial strata of North America’s Western Interior Basin record an exceptional peak in the diversification of fossil vertebrates in the Campanian, which has been termed the ‘zenith of dinosaur diversity’. The wide latitudinal distribution of rocks and fossils that represent this episode, spanning from northern Mexico to the northern slopes of Alaska, provides a unique opportunity to gain insights into dinosaur paleoecology and to address outstanding questions regarding faunal provinciality in connection to paleogeography and climate. Whereas reliable basin-wide correlations are fundamental to investigations of this sort, three decades of radioisotope geochronology of various vintages and limited compatibility has complicated correlation of distant fossil-bearing successions and given rise to contradictory paleobiogeographic and evolutionary hypotheses. Here we present new U–Pb geochronology by the CA-ID-TIMS method for 16 stratigraphically well constrained bentonite beds, ranging in age from 82.419 ± 0.074 Ma to 73.496 ± 0.039 Ma (2σ internal uncertainties), and the resulting Bayesian age models for six key fossil-bearing formations over a 1600 km latitudinal distance from northwest New Mexico, USA to southern Alberta, Canada. Our high-resolution chronostratigraphic framework for the upper Campanian of the Western Interior Basin reveals that despite their contrasting depositional settings and basin evolution histories, significant age overlap exists between the main fossil-bearing intervals of the Kaiparowits Formation (southern Utah), Judith River Formation (central Montana), Two Medicine Formation (western Montana) and Dinosaur Park Formation (southern Alberta). Pending more extensive paleontologic collecting that would allow more rigorous faunal analyses, our results support a first-order connection between paleoecologic and fossil diversities and help overcome the chronostratigraphic ambiguities that have impeded the testing of proposed models of latitudinal provinciality of dinosaur taxa during the Campanian.

## Introduction

Continental sedimentary successions are important archives of terrestrial flora and fauna, as well as the paleoenvironmental conditions that dominated the continents in the geologic past. However, establishing the tempo and patterns of evolution in relation to documented climatic and geologic changes is limited by the ability to precisely date and correlate fossil-bearing strata. Understanding the depositional history of continental records in absolute time thus has multiple scientific merits: (1) it makes it possible to place geographically scattered fossil occurrences in a proper chronostratigraphic framework as a basis for paleobiologic interpretations, (2) it allows the construction of time-calibrated records of terrestrial paleoenvironmental change (e.g., climatic and geologic) in which possible links to the coeval biotic evolution can be explored, (3) it facilitates correlation of the continental biotic records to the marine biostratigraphy upon which the geologic time scale has been built and, (4) it enables transcontinental correlation of biotic and paleoenvironmental records as a basis for assessing global evolutionary and paleoecologic models. Unravelling the detailed depositional history of continental successions is nevertheless challenging because of the inherently discontinuous nature of non-marine sedimentation in space and time and the paucity of diagnostic, age-specific fossils in this setting. As such, radioisotopic geochronology plays a crucial role in constructing reliable chronostratigraphic frameworks for continental successions by providing temporal tie lines independent of often equivocal litho-, bio- or magneto-stratigraphic correlations.

**Figure 1:**
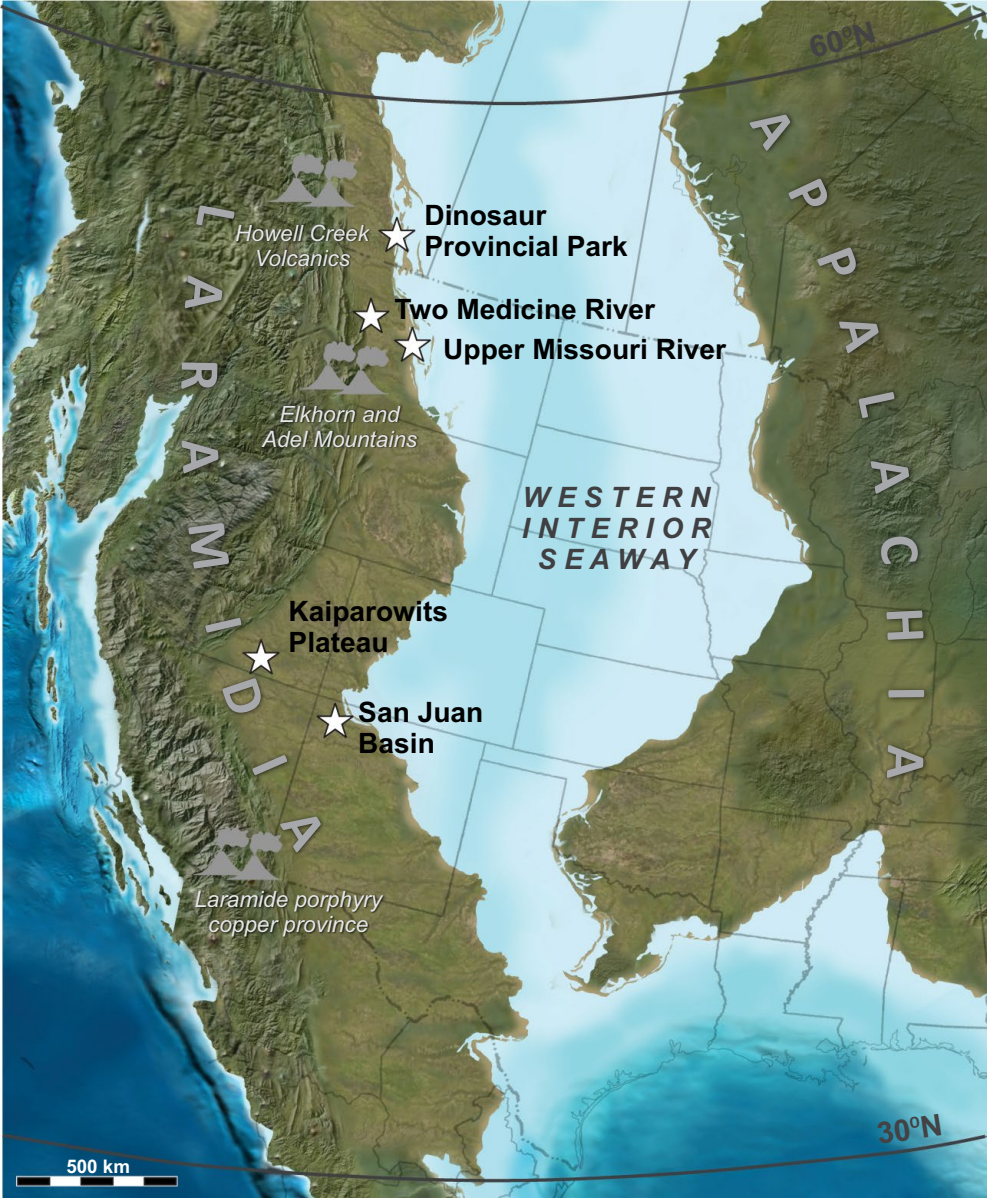
Paleogeographic reconstruction of the North American continent in the late Campanian illustrating the areal expanse of the Western Interior Seaway separating the Laramidia and Appalachia landmasses, after Blakey^33^. Our study locations are marked by stars. Potential centers of coeval arc volcanism are also indicated. Source map © 2022 Colorado Plateau Geosystems Inc.

The Western Interior Basin (WIB) of North America (Fig. 1) contains extensive exposures of Upper Cretaceous rocks preserving a spectacular fossil record. In particular, the Campanian stage (ca. 84–72 Ma) is unparalleled for exposures of vertebrate-bearing continental strata on the northern continents and has been termed the ‘zenith’ of dinosaur diversity^1–4^. The cause(s) of the inferred Campanian diversification remains poorly understood and related evolutionary models have not been adequately tested. A subject of particular debate is whether or not the Campanian dinosaur record of the WIB exhibits a latitudinal provinciality^5–7^. Moreover, whether or not the remarkable faunal richness of the Campanian is a taphonomic/preservational artefact or signifies a true increase in biologic diversity remains a matter of debate^8,9^. Addressing these questions requires a thorough understanding of environmental controls on fossil preservation, sampling bias in collections, and high-resolution correlations between outcrop areas along nearly 2000 km of strike, as well as control on the tempo of climate and biological changes.

Biotic evolution during the Campanian transpired against a background of profound environmental change, including a global increase in atmospheric temperatures and an abrupt rise in sea levels that flooded low lying continental regions^10–12^. The Campanian was also a time of widespread explosive volcanism in Montana (Elkhorn Mountain and Adel volcanics) and British Columbia (Howell Creek volcanics), but also farther to the south in Texas, southern Arizona, southern New Mexico and northern Mexico^13–18^ (Fig. 1). Easterly Campanian trade winds transported volcanic ash from western and southern sources into the WIB^19–22^, depositing numerous ash beds that are intercalated throughout the stratigraphic succession. During the last 25 years, ^40^Ar/^39^Ar (and K–Ar) geochronology has been widely utilized to date Upper Cretaceous strata throughout the WIB, in particular the bentonite (devitrified and altered volcanic ash) horizons, to constrain ammonite biozones^23,24^. Similarly, a number of continental sedimentary successions, including the Campanian Two Medicine^25^, Judith River^26^, Dinosaur Park^27^, Kaiparowits^28^, Kirtland, and Fruitland^29^ formations were targeted for geochronology because of their rich vertebrate fossil records. This has facilitated correlation of stratigraphic sections and faunas across the marine and non-marine WIB. However, this and subsequent bodies of work were conducted over more than three decades in different laboratories and by implementing different analytical protocols, resulting in many vintages of geochronologic data that in many cases lack compatibility. This has hampered a full interrogation of the WIB paleontologic, sedimentologic, and paleoclimatologic records. Radioisotopic geochronologic techniques have improved dramatically over the past decade and it is now possible to build age models for Cretaceous rocks at the millennial to decamillennial scales^30–32^. This, in turn, affords new opportunities to examine trends in geological and paleontological records at high resolution.

Here we present a set of internally consistent geochronologic data from five of the most richly fossiliferous Campanian continental successions—namely the Dinosaur Park, Two-Medicine, Judith River, Kaiparowits and Fruitland/Kirtland formations. These units are separated by as much as 1600 km and 15 degrees of paleolatitude along the WIB forelands. The geochronology presented in this paper is unique compared to previous studies in that it is based entirely on high-precision U–Pb analyses of bentonitic zircon by the CA-ID-TIMS method and that it employs the latest, community-wide, analytical practices and protocols. Our new geochronology provides bed-level correlation of bentonites and fossil-rich stratigraphic intervals across the study areas and serves as a foundation for basin-wide study of the Campanian dinosaur record. The results place in context a large and growing body of paleontologic, sedimentologic and paleoecologic data that has been collected throughout the WIB, providing an opportunity to evaluate temporal relationships among well studied floras and faunas and test hypotheses relating to latitudinal provinciality across Laramidia.

## Laramidian stratigraphy and geological setting

The meridional Western Interior Seaway (WIS) covered vast areas of North America from Alaska to Mexico^33^, bisecting the continent during much of the Cretaceous Period and isolating a narrow western landmass (Fig. 1) referred to as Laramidia^34^. The shorelines of the WIS transgressed and regressed throughout its history in response to climatic and/or tectonic drivers. The Cretaceous alluvial and coastal plain deposystems that formed along the western shores of this vast epicontinental seaway archive a rich record of well-preserved vertebrates, invertebrates and plants. A long history of fossil collection (> 100 years) has rendered Laramidia a cornerstone of Mesozoic paleobiogeographic research on dinosaurs and numerous other land floral and faunal groups.

**Figure 2:**
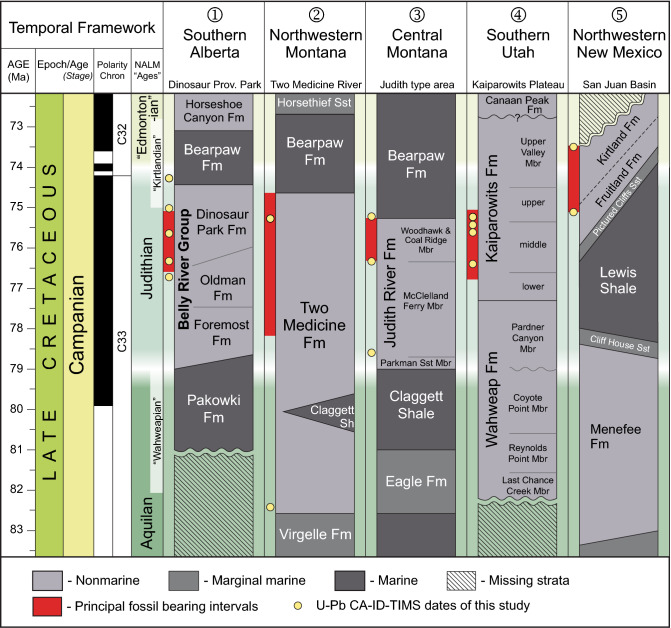
Correlation chart of rock formations and principal vertebrate-bearing continental deposits of Campanian age in the Western Interior Basin, modified from Roberts et al.^105^ with new data^86,114,126^ and the results of this study. Shown in red are the calibrated temporal ranges of principal fossil-bearing intervals (excluding uncertainties). Fm, Formation; Mbr, Member; NALM, North American Land Mammal; Sh, shale; Sst, sandstone.

Continental Upper Cretaceous strata are well-preserved across western North America, from Alaska to Mexico (Figs. 1, 2). Not surprisingly, a census of Mesozoic vertebrate fossil occurrences in North America using the Paleobiology Database (paleobiodb.org/) reveals a significant spike in fossil preservation during the Late Cretaceous, with greatest diversity and fossil abundance linked to the Campanian and Maastrichtian stages^1,4^. A closer inspection of the database demonstrates that the majority of these fossils come from relatively few stratigraphic units and indeed, represent fairly narrow temporal windows. The second half of the Campanian (ca. 77–72 Ma) is arguably the richest interval within this time frame and is highlighted by especially large curated collections from Alberta, Montana, Utah, and New Mexico. The bulk of the fossil occurrences are known from a few key regions of the WIB, including Alberta (Oldman and Dinosaur Park formations), Montana (Two Medicine and Judith River formations), Utah (Wahweap and Kaiparowits formations), and New Mexico (Kirtland and Fruitland formations) (Figs. 1, 2).

### Dinosaur Provincial Park, Alberta, Canada

The Upper Cretaceous Belly River Group of Alberta has yielded arguably the most diverse and best sampled Cretaceous terrestrial fossil assemblage in the world^35^. It consists in ascending order of the Foremost, Oldman, and Dinosaur Park formations (Fig. 2), which are overlain by the marine shales of the Bearpaw Fm.^36,37^. The thickness of the three formations at Dinosaur Provincial Park (based on both surface and subsurface data) are approximately 170 m, 40 m, and 70 m, respectively^37^, with the Oldman and Dinosaur Park formations locally exhibiting thicknesses up to ca. 50 and 80 m, respectively, due to differential compaction on stacked sandstones and local paleochannel relief at the base of the Dinosaur Park Fm.

The paralic to non-marine (alluvial) clastic sediments of the Foremost Formation (Fm.) are organized into progradational to aggradational stratigraphic packages that reflect an overall regressive WIS trend^37^. Two prominent coal zones (McKay and Taber) are present in the lower and upper portions of the formation, respectively. The overlying Oldman Fm. is part of an east-northeast-expanding clastic wedge originating in northwest Montana that records maximum regression of the WIS into south-central Saskatchewan^37^. It consists of a variety of fluvial and floodplain facies deposited seasonally across a relatively well-drained landscape. A subsequent transgressive phase of the WIS is recorded in the overlying bentonite-rich, alluvial-to-paralic facies of the Dinosaur Park Fm. The stratigraphically lower one-half of the formation is dominated by paleochannel deposits that are unusually rich in bone-beds and articulated-to-associated skeletons of large dinosaurs^35,37^. The formation culminates upsection in the Lethbridge Coal Zone (LCZ), a package of tidally influenced wetland-to-shoreline deposits (including up to four coal beds)^38^ that interfinger with—and are overlain by—marine shales of the Bearpaw Fm.

The Oldman and Dinosaur Park formations are well exposed and extensively sampled at Dinosaur Provincial Park, yielding many hundreds of articulated and associated dinosaur specimens, and thousands of isolated elements from more than 140 species of vertebrates^35,39–46^. Fossil collection in the Park has been conducted for more than a century, although a viable stratigraphic framework for the fossils is a more recent development^35,45,47,48^. Radioisotopic geochronology in the Park extends back to the early 1990’s, but was developed in piecemeal fashion^27,36^. Seven stratigraphically discrete bentonite horizons have been documented^49^, with ^40^Ar/^39^Ar ages that range from 76.5 ± 1.0 Ma to 74.9 ± 0.2 Ma (2σ internal uncertainties hereafter, unless specified otherwise).

### Western and central Montana, USA

To the south of the international border in Montana, the richly fossiliferous Two Medicine and Judith River formations form an eastward thinning clastic wedge of alluvial, coastal plain, and shallow marine strata deposited during the Campanian^50^. The strata of the Two Medicine Fm. crop out in northwestern Montana and represent the more proximal alluvial plain environments of the Two Medicine-Judith River wedge. The sedimentology and taphonomy of the unit are consistent with accumulation under seasonal, semi-arid conditions^50–52^. The alluvial deposits of the Two Medicine Fm. are truncated by the Cordilleran thrust front to the west and are separated from the more distal outcrop belt of the correlative Judith River Fm. to the east by the Sweetgrass arch.

The ca. 550-m-thick Two Medicine Fm. is well known for its dinosaur fauna, and especially the exquisite preservation of dinosaur nests and hatchlings^51,53–57^. Vertebrate fossil collection in the unit began more than a century ago, and Two Medicine deposits have yielded one of the best documented Cretaceous vertebrate assemblages in the world. Rogers et al.^25^ undertook a comprehensive geochronologic study in the Two Medicine Fm. based on ^40^Ar/^39^Ar analyses of primarily plagioclase and biotite (and one sanidine), and reported dates from five bentonite beds that ranged from 80.00 ± 0.56 Ma to 74.08 ± 0.19 Ma , spanning most of the formation in its type area. Subsequent ^40^Ar/^39^Ar work (sanidine and plagioclase) by Foreman et al.^58^ added an additional date of 77.52 ± 0.38 Ma to a bentonite intercalated in the approximate middle of the formation. Varricchio et al.^59^ reported ^40^Ar/^39^Ar analyses (plagioclase and biotite), as well as U–Pb zircon geochronology by the SIMS method from multiple bentonites associated with a prominent hadrosaur bone bed in the southern Two Medicine outcrop belt. Their geochronology from strata attributed to the uppermost Two Medicine Fm. resulted in an ^40^Ar/^39^Ar plateau age of 75.92 ± 0.64 Ma, whereas the youngest measured single zircon U–Pb date was 74.0 ± 1.8 Ma^59^. The published radioisotopic geochronology from Two Medicine Fm. spans much of the spatial and temporal distribution of the formation from ca. 80 Ma to 74 Ma, with highly variable 2σ internal uncertainties (± 0.19 to ± 1.8 Myr) and accuracies (Supplementary Table S1). A temporally calibrated stratigraphy with adequate resolution between key fossil localities has never been compiled.

The ca. 180-m-thick Judith River Fm. is widely exposed along the Missouri River corridor in north-central Montana, within the confines of the Upper Missouri River Breaks National Monument. Stratigraphic research during the mid-to-late nineteenth and early twentieth centuries on the age and correlation of the Judith River Fm. was instrumental in resolving the basic stratigraphy of the Western Interior Cretaceous section^60–66^. The unit is also significant to the history of vertebrate paleontology, with some of the first skeletal remains of dinosaurs described from North America recovered from the Judith River strata near the confluence of the Judith and Missouri Rivers^67–70^. The Judith River Fm. has continued to be a major focus of paleontological research to the present day^71–82^.

Among the earliest ^40^Ar/^39^Ar geochronology from the Campanian of the WIB were the sanidine analyses of Goodwin and Deino^26^ from two bentonite beds associated with the Taber Coal Zone of the lower Judith River Fm. in Kennedy Coulee, northern Montana. More recently, Rogers et al.^83^ reported three ^40^Ar/^39^Ar sanidine ages from the Judith River Fm. and overlying strata in its type area in central Montana, ranging from 76.24 ± 0.36 Ma to 75.21 ± 0.24 Ma.

### Kaiparowits Plateau, Utah, USA

The Campanian strata of the Kaiparowits Plateau encompass the Wahweap and Kaiparowits formations, which were deposited in the southern part of the WIB. The Wahweap Fm. is a ca. 400-m-thick succession of channel sandstones and floodplain mudstones of fluvial and estuarine origin deposited by meandering rivers under a seasonal climate^84–86^. Its sedimentation was largely influenced by transgression of the WIS until a tectonically-driven drop in the base level led to a change in depositional setting and sediment source reflected in its uppermost Pardner Canyon (capping sandstone) Member. This change is represented at the member’s lower boundary by prominent channel incisions into the underlying strata^87^.

The overlying 1005-m-thick Kaiparowits Fm.^88,89^ represents extensive flood basin pond, lake, and river deposition on a low-relief alluvial plain characterized by a warm, subhumid paleoenvironment^90^. High volcanic input and rapid rock accumulation rates due to active tectonic subsidence characterizes this unit^28,91,92^. The Kaiparowits Fm. preserves an abundant and remarkably diverse flora and fauna ranging from invertebrates to large vertebrates^7,93,94^.

Documentation of the fossil vertebrates and stratigraphy of the Kaiparowits and Wahweap formations extends back to the 1930’s, although very little work transpired in this region due to its remoteness until the 1980’s, when the importance of the mammalian and other microfossils from these units was recognized^95,96^. Renewed interest in macrofossils from the Kaiparowits Fm. began in the early 2000s and has led to many new discoveries of vertebrate, invertebrate, and plant macrofossils, including many new dinosaur species. As a result of these efforts, the fossil record of southern Utah now eclipses that of most other Cretaceous formations in North America in terms of diversity and abundance^7,97–104^.

A high resolution stratigraphic record has been developed in parallel to vertebrate fossil exploration in the Kaiparowits Fm., which has resulted in the identification of at least 10 discrete bentonite horizons^28,105^. ^40^Ar/^39^Ar dating of sanidine was used to determine depositional ages for five of these bentonites, which ranged from 75.97 ± 0.36 Ma to 74.69 ± 0.36 Ma.

### San Juan Basin, New Mexico, USA

The non-marine Campanian succession of the San Juan Basin (northwestern New Mexico and southwestern Colorado) consists of the Fruitland Fm. and the overlying Kirtland Fm.^106^, with a maximum combined thickness in excess of 300 m in portions of the basin. The coal-bearing Fruitland Fm. consists of sandstones, siltstones, and carbonaceous shales that were deposited in a nearshore swamp setting on top of the regressive shoreline beds of the Pictured Cliff Sandstone^107^. It grades upward (and laterally) into the predominantly fluvial sandstones and shales of the Kirtland Fm., which in turn is overlain unconformably by the early Paleocene Ojo Alamo/Animas Fm.^108^. The strongly transitional and time-transgressive nature of the contact between the Fruitland and Kirtland formations complicates their lithostratigraphic division across the basin^107^.

Vertebrate fossil collections from the Upper Cretaceous strata of the San Juan Basin extend back over 100 years, with early collecting efforts led by Hay^109^ and Gilmore^110,111^. Since then, thousands of specimens have been collected, with the richest stratigraphic intervals characterized by two main “local” faunas, namely the Hunter Wash and Willow Wash local faunas^112,113^.

The earliest published radioisotopic dates for the Fruitland and Kirtland formations are those of Fassett and Steiner^29^, who reported ^40^Ar/^39^Ar sanidine dates for five bentonites distributed throughout the Fruitland-Kirtland succession. These ^40^Ar/^39^Ar ages (compiled in Fassett^107^) ﻿range from 75.56 ± 0.41 Ma (Huerfanito Bentonite Bed) at the base of the Fruitland Fm to 73.04 ± 0.25 Ma (Ash J) at the top of the Kirtland Fm., although most are within error of each other, limiting their utility for fine temporal resolution. More recent ^40^Ar/^39^Ar geochronology from three bentonites (DEP, Ash 2 and Ash J) in this succession have resulted in improved precision and accuracy^114^.

## Previous radioisotopic age framework

Early radioisotopic age constraints on the Campanian formations of the WIB consisted of K–Ar analyses of biotite and feldspar from interbedded bentonites^115,116^, with assumed uncertainties on the order of ± 5%, and these analyses are not discussed further here. Biotite ages, in particular, are considered of questionable accuracy by modern standards because of the suspected alteration-related ^39^Ar recoil and redistribution effects in multigrain analyses^117^. The advent of the ^40^Ar/^39^Ar isotopic method greatly improved the internal precisions of measured dates by allowing the parent and daughter isotopes to be measured on the same sample aliquot by the same technique. Early ^40^Ar/^39^Ar geochronology of bentonites from northern Montana and southern Alberta confirmed the Campanian ages of the Judith River Fm.^26^, as well as those of the Oldman and Dinosaur Park formations^27,36^. Subsequent systematic geochronologic studies were aimed at constructing chronostratigraphies for the Two Medicine Fm.^25^ and Judith River Fm.^83^ in Montana, the Kaiparowits Fm. in Utah^28^ and the Kirtland/Fruitland formations in New Mexico^29,107^. These ^40^Ar/^39^Ar data were produced in three different laboratories in the course of nearly three decades (Supplementary Table S1), during which time significant revisions were made to the decay constant of ^40^ K used for age calculation^117,118^. In addition, various mineral standards were co-irradiated with samples as neutron flux monitors (e.g., Mmhb-1 hornblende^119﻿^) p﻿rior to the general adoption of the Fish Canyon Tuff (FCT) sanidine, whose independent age has been revised frequently (e.g., 27.84 Ma^120^, 28.02 Ma^121^, 28.201 Ma^122﻿^). This incompatibility of ^40^Ar/^39^Ar geochronology of different vintages limits the accuracy of published bentonite ages beyond the typically reported uncertainties of ± 0.2–0.5% (see Supplementary Table S1). In order to improve the compatibility of legacy data, various authors have attempted to recalculate previously published ages from the WIB with modern mineral standard ages and/or decay constants^105,114,123^. However, these recalculations do not account for dissimilar protocols for Ar gas extraction or mass spectrometric data acquisition and data reduction employed by different laboratories at different times. This ^40^Ar/^39^Ar ‘interlaboratory bias’ was estimated to be larger than ± 2% by Min et al.^117^, which exceeded the reported analytical uncertainties. Although ongoing intercalibration efforts under the EARTHTIME initiative^124^ promise a significant reduction in bias among the participating laboratories, its present magnitude remains difficult to quantify. Supplementary Table S1 summarizes the previously published geochronology with available analytical data from the upper Campanian of the WIB, without any further recalculation. It is notable that the most recent astronomically tuned calibration of the FCT sanidine standard recommends a revised age of 28.176 ± 0.023 Ma for this standard^125^.

About a dozen U–Pb zircon dates have previously been reported from the Campanian of the WIB (Supplementary Table S1) and all but two are microbeam U–Pb analyses of detrital zircons^59,91^. The only two published CA-ID-TIMS bentonite ages were from the lower portion of the Kaiparowits Fm. in Utah^105^ and the basal Bearpaw Fm. in southern Alberta^126^.

## New U–Pb CA-ID-TIMS geochronology

**Figure 3:**
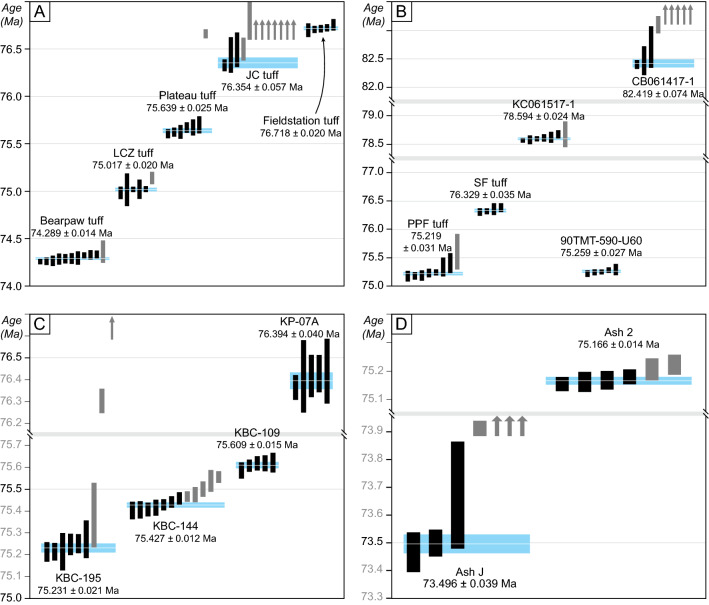
Ranked age plot of analyzed zircons from Campanian ash beds of the Western Interior Basin. (**A**) Dinosaur Provincial Park, southern Alberta, Canada; (**B**) Two Medicine and Judith River formations, Montana; (**C**) Kaiparowits Formation, southern Utah; (**D**) Kirtland and Fruitland formation, San Juan Basin, New Mexico. Vertical bars are individual zircon analyses with their 2σ analytical uncertainty; black bars are analyses used in age calculation. Arrows represent the analyses that plot outside the diagram. Blue band signifies the weighted mean age with its 95% confidence level (2σ) internal uncertainty (*X*). See Table 1 for bentonite and age information, and Supplementary Table S2 for complete U–Pb isotopic data. JC, Jackson Coulee; LCZ, Lethbridge Coal Zone; PPF, Power Plant Ferry; SF, Stafford Ferry.

Meaningful paleobiologic analyses of fauna associated with the zenith of dinosaur diversity in the Campanian requires a set of internally consistent radioisotopic ages of wide geographic distribution upon which high resolution chronostratigraphic frameworks can be constructed and unambiguous temporal correlations across the WIB successions can be made. A total of 118 single-zircon U–Pb analyses were carried out by the CA-ID-TIMS method on 16 bentonite samples (Supplementary Figures S1 and S2) from seven lithostratigraphic units across the WIB. The selected units are the Oldman, Dinosaur Park and Bearpaw formations in southern Alberta, the Judith River, Two Medicine and Bearpaw formations in Montana, the Kaiparowits Fm. in southern Utah, and the Kirtland/Fruitland formations in New Mexico (Fig. 3 and Table 1). The application of EARTHTIME isotopic tracers, data reduction software and recommended analytical protocols for CA-ID-TIMS geochronology assures consistency and effective mitigation of interlaboratory bias in the acquired data.

**Table 1:** Summary of calculated U–Pb ages and their uncertainties.

Sample	Tuff name^a^	Formation/Member^b^	Latitude^c^	Longitude^c^	Elevation (m)^d^	^206^Pb/ ^238^U		Uncertainty (2σ)^e^			MSWD^f^	*n*^g^	*#*
						Age (Ma)		*X*	*Y*	*Z*			
*Dinosaur Park, Alberta*													
IL082717-1	Bearpaw	Bearpaw	50° 45′ 21.0″	− 111° 22′ 52.0″	83.25	74.289		0.014	0.024	0.083	2.0	10	11
LCZ2	LCZ	Dinosaur Park	50° 49′ 26.3″	− 111° 19′ 27.4″	61.50	75.017		0.020	0.028	0.085	0.31	5	6
CD082717-1	Plateau	Dinosaur Park	50° 44′ 46.9″	− 111° 29′ 18.7″	36.00	75.639		0.025	0.032	0.087	1.3	6	7
JC082817-1	JC	Dinosaur Park	50° 45′ 05.5″	− 111° 24′ 33.4″	1.25	76.354		0.057	0.061	0.10	1.9	3	12
FS082717-1	Fieldstation	Oldman	50° 45′ 33.1″	− 111° 31′ 04.1″	− 5.50	76.718		0.020	0.029	0.087	0.58	5	5
*Central and northern Montana*													
PPF1-03	PPF	Bearpaw	47° 43′ 29.2″	− 108° 56′ 36.7″	180	75.219		0.031	0.046	0.093	1.7	7	9
ST1-03	SF	Judith River/CR	47° 45′ 37.2″	− 109° 19′ 46.9″	84	76.329		0.035	0.043	0.092	0.92	4	4
KC061517-1	–	Judith River/MCF	48° 56′ 58.5″	− 110° 36′ 09.3″	31	78.594		0.024	0.032	0.090	1.4	6	7
*Western Montana*													
90TMT-590	–	Two Medicine/upper	48˚29′ 50.0″	− 112˚36′ 16.4″	460	75.259		0.027	0.034	0.087	0.59	4	4
CB061417-1	–	Two Medicine/lower	48° 31′ 33.1″	− 112° 17′ 22.3″	15.5	82.419		0.074	0.086	0.12	1.4	3	8
*Kaiparowits Plateau, Utah*													
KBC-195	–	Kaiparowits/upper	37° 38′ 12.7″	− 111° 50′ 41.7″	612	75.231		0.021	0.038	0.089	0.73	6	9
KBC-144	–	Kaiparowits/middle	37° 37′ 59.9″	− 111° 50′ 51.3″	498	75.427		0.012	0.023	0.084	1.80	7	12
KBC-109	–	Kaiparowits/middle	37° 37′ 46.8″	− 111° 51′ 18.7″	420	75.609		0.015	0.025	0.085	0.62	5	5
KP-07A	–	Kaiparowits/Middle	37° 26′ 11.9″	− 111° 41′ 51.8″	180	76.394		0.040	0.045	0.093	0.60	5	5
*San Juan Basin, New Mexico*													
SJB-1801	Ash J	Kirtland	36° 21′ 28.2″	− 108° 07′ 55.7″	384	73.496		0.039	0.046	0.091	2.0	3	7
SJB-1802	Ash 2	Fruitland	36° 17′ 48.2″	− 108° 13′ 36.8″	136	75.166		0.014	0.025	0.084	0.69	4	6

^a^JC, Jackson Coulee; LCZ, Lethbridge Coal Zone; PPF, Power Plant Ferry; SF, Stafford Ferry.

^b^CR, Coal Ridge Member; MCF, McClelland Ferry Member.

^c^Latitude/Longitude relative to WGS 84 datum. For San Juan Basin samples they are estimated from Fassett^107^ maps locations.

^d^Stratigraphic elevation above the formation boundary, where measured.

^e^*X*-internal (analytical) uncertainty in the absence of all external or systematic errors; *Y*-incorporates *X* and the U–Pb tracer calibration error; *Z*-includes *X* and *Y*, as well as the uranium decay constant errors of Jaffey et al.^163^.

^f^MSWD, mean square of weighted deviates.

^g^*n*, number of analyses included in the calculated weighted mean date out of total number of analyses (#).

Details of analytical procedures including zircon chemical abrasion and age interpretations are described in the Methods. Bentonite ages are derived from weighted mean ^206^Pb/^238^U dates of statistically coherent zircon analyses and are reported at the 95% confidence interval in the format ± *X*/*Y*/*Z* Ma, where *X* is the internal error based on analytical uncertainties only, *Y* includes *X* and the tracer calibration uncertainty, and *Z* includes *Y* plus the ^238^U decay constant uncertainty (Table 1). For comparing CA-ID-TIMS analyses that used the same U–Pb isotopic tracer, including the age-stratigraphic models discussed below, the systematic uncertainties (*Y* and *Z*) can be ignored and only the internal age uncertainties (*X*) are considered. However, meaningful comparisons between U–Pb and ^40^Ar/^39^Ar ages require external uncertainties (including decay constant errors) to be taken into account. Bayesian age-stratigraphic modelling was employed to extrapolate ages with objective uncertainties for stratigraphic levels of interest (e.g., fossil beds) in between dated horizons (Supplementary Table S3). The model produces larger (asymmetric) age uncertainties with distance from dated bentonites in order to account for possible discontinuities or changes in the rock accumulation rate.

The Cretaceous time scale calibration of Gale et al.^127^ is followed here, in which the base and top of the Campanian Stage is placed at 83.7 ± 0.5 Ma and 72.2 ± 0.2 Ma, respectively. As the biostratigraphically defined base of the Campanian Stage based on WIB ammonite zonation lacks reliable age constraints, extrapolation of the boundary to the base of the geomagnetic polarity Chron 33R is presently used as an indirect calibration. The base of the Maastrichtian is better defined by belemnite, inoceramid and ammonite biostratigraphy and calibrated by ^40^Ar/^39^Ar geochronology^127^. The primary focus of this study has been the Campanian rocks deposited after ca. 78 Ma.

## Results

Our CA-ID-TIMS U–Pb geochronology provides a set of high-precision ages for 16 Campanian tuffs across the WIB based on statistically robust weighted mean ^206^Pb/^238^U dates derived from three to ten overlapping zircon analyses (within *X*) from each sample. All weighted mean dates obey the stratigraphic superposition and thus represent with confidence the eruption ages of the corresponding ash beds; higher confidence is associated with larger number of overlapping analyses (*n*) and fewer outliers. No outlier has been excluded from date calculation for being too young. All of the dated samples are high-purity bentonites interpreted as direct products of volcanic ash fall deposits, whose eruption ages within uncertainty represent good approximations for the age of sedimentary deposition. Complete U–Pb isotopic data are given in Supplementary Table S2; the calculated ages and the breakdown of their uncertainties are listed in Table 1 and illustrated in Fig. 3.

The new U–Pb geochronology has an average internal uncertainty (*X*) of ± 26 kyr (fully propagated average uncertainty of *Z* =  ± 88 kyr), which is an order of magnitude improvement in precision over the latest published ^40^Ar/^39^Ar geochronology from the WIB (excluding decay constant errors: Supplementary Table S1). This allows temporal resolution of closely spaced ash beds with eruption frequency as high as ca. 40 kyr and thus their unambiguous correlation over 1600 km of latitudinal distance.

The high-precision geochronology and Bayesian age-stratigraphic models of this study are used to place robust age constraints on select fossiliferous intervals of the WIB Campanian formations (see below). These intervals are defined as those containing the highest concentrations of vertebrate fossils based on historical discoveries and including fauna that have formed the basis of dinosaur provinciality hypotheses. They by no means incorporate all known Campanian fossil beds from the corresponding units. Also excluded from discussions are earlier Campanian fossil assemblages (e.g., those of the Oldman Fm. in Alberta and the Wahweap Fm. in Utah), which fall outside the scope of this study.

### Dinosaur Provincial Park, Alberta, Canada

**Figure 4:**
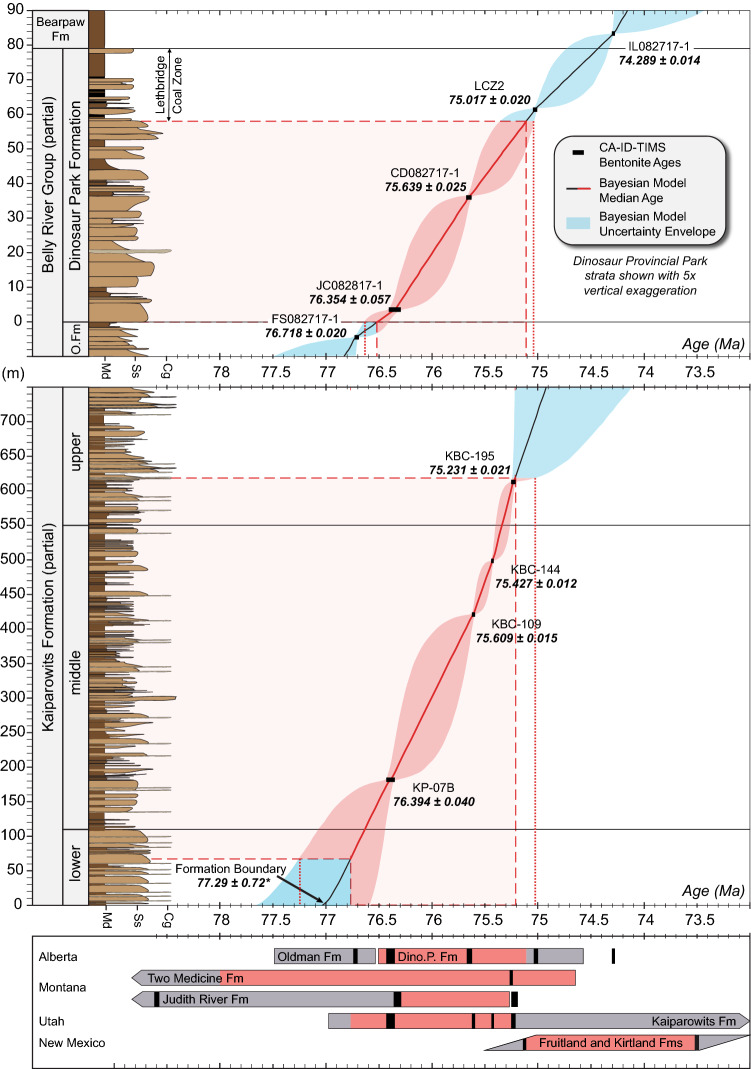
Lithostratigraphy, U–Pb geochronology and Bayesian age-stratigraphic models for the Dinosaur Park (top) and Kaiparowits (middle) formations. Dinosaur Park and Kaiparowits stratigraphy after Eberth and Hamblin^36^ and Roberts et al.^105^, respectively. Red shading corresponds to the principal fossil-bearing intervals and their model ages, dotted lines incorporate the model age uncertainties. Bottom panel illustrates the interformational correlation of fossil intervals based on this study. ^*^Boundary age from Beveridge et al.^86^.﻿

U–Pb ages for five bentonites from well-correlated stratigraphic sections within the Park and its vicinity are used to construct a high resolution age-stratigraphic model for the Dinosaur Park Fm. (Fig. 4 and Supplementary Fig. S2-A). The ages (Table 1) range from 76.718 ± 0.020 Ma (Fieldstation tuff, Oldman Fm.) to 74.289 ± 0.014 Ma (Bearpaw tuff, Bearpaw Fm.), spanning 89 m of strata. The resulting age model places the lower and upper boundaries of the Dinosaur Park Fm. at 76.470 + 0.14/ − 0.084 Ma and 74.44 + 0.30/ − 0.11 Ma, respectively. This extends the duration of the formation to 2.03 ± 0.18 Myr from the Eberth (2005) estimate of 1.7 Myr. The main vertebrate fossil-bearing stratigraphic interval of the formation is constrained between 76.61 and 75.04 Ma (range incorporates 95% confidence intervals).

### Western and central Montana, USA

U–Pb geochronology from the Two Medicine Fm. includes a bentonite age of 82.419 ± 0.074 Ma (sample CB061417-1) from 15.5 m above the base of the formation on the eastern flank of Cut Bank Creek, about 12 km south of Cut Bank, Glacier County, Montana (Supplementary Fig. S2-C). Another bentonite (sample 90TMT-590) from approximately 40 m below the top of the formation in its type area produced a U–Pb age of 75.259 ± 0.027 Ma. The former is so far the oldest reported age from the Two Medicine Fm., extending its age into the lower Campanian and indicating more than 7 Myr of deposition for the formation. The principal fossil-rich interval of the Two Medicine Fm. starts below its lacustrine carbonate facies (ca. 290 m above the formation base) in its type area, and includes the many fossil discoveries in the classic Egg Mountain locality. Pending a more comprehensive age model for the formation, our limited U–Pb geochronology broadly constrains its fossil-rich interval between ca. 78.2 Ma and 74.6 Ma.

**Figure 5:**
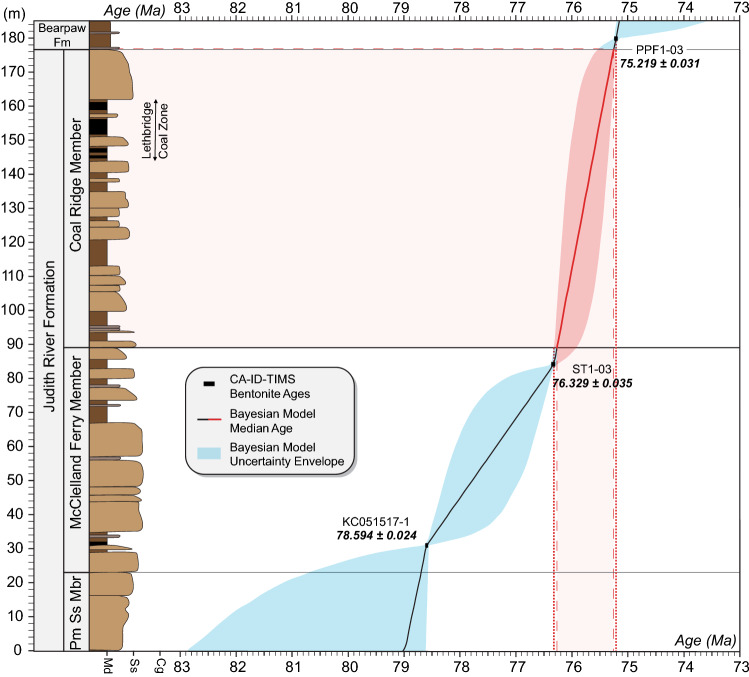
Lithostratigraphy (after Rogers et al.^83^), U–Pb geochronology and Bayesian age-stratigraphic model for the Judith River Formation in its type area. Pm Ss Mbr, Parkman Sandstone Member. Red shading corresponds to the principal fossil-bearing interval and its model age, dotted lines incorporate the model age uncertainties. Note that the single U–Pb age from Kennedy Coulee (KC051517-1) has been projected onto the type stratigraphy (see text for explanation).

Our U–Pb geochronology from key stratigraphic horizons of the Judith River and Bearpaw formations (Table 1 and Fig. 3) includes an age of 78.594 ± 0.024 Ma from a bentonite (sample KC061517-1) within Coal Marker A bed at Kennedy Coulee, Hill County, Montana (Supplementary Fig. S2-B), as well as 76.329 ± 0.035 Ma (Stafford Ferry tuff, McClelland Ferry Member) and 75.213 ± 0.021 Ma (Powerplant Ferry tuff, Bearpaw Fm.) from the Judith River Fm. type area in the Upper Missouri River Breaks National Monument. Kennedy Coulee is located about 170 km to the north-northwest of the type area and its prominent coal beds have been linked to the widespread Taber Coal Zone of southern Alberta^25^, making them stratigraphic correlatives to the coal beds of the lower McClelland Ferry Member in the type area. The three U–Pb ages can be used to construct an age-stratigraphic model (Fig. 5) that places the top of the Judith River Fm. (base of the Bearpaw Fm.) at 75.255 + 0.27/ − 0.039 Ma in the type area. Accordingly, the base and top of the McClelland Ferry Member are constrained to 78.70 + 2.0/ − 0.11 Ma and 76.269 + 0.052/ − 0.34 Ma, respectively. However, there is considerable uncertainty associated with the lower age constraint as the Taber coal zone is expected to be time transgressive^36^ and is likely to shift lower in stratigraphy towards Kennedy Coulee. Therefore, the ca.78.70 Ma age for the member boundary should be considered a maximum estimate. The main concentration of vertebrate fossils in the formation occurs within its Coal Ridge Member^83^, which is very well constrained to between 76.32 and 75.22 Ma (Fig. 5).

### Kaiparowits Plateau, Utah, USA

Weighted mean U–Pb dates of this study from the Kaiparowits Fm. (Table 1) incorporate four bentonites from its middle and upper (informal) units, three of which are in succession from the Kaiparowits Blues type section (Supplementary Fig. S2-D), whereas the stratigraphically lowest one is from the Horse Mountain section^90^. They range in age from 76.394 ± 0.04 Ma (Sample KP-07A, middle unit) to 75.231 ± 0.021 Ma (Sample KBC-195, upper unit), encompassing 432 m of strata (Fig. 4). The resulting age-stratigraphic model places the middle unit of the formation between 76.63 + 0.44/ − 0.19 Ma and 75.364 + 0.046/ − 0.085 Ma, with the primary vertebrate fossil-producing interval of the formation constrained to between 77.24 and 75.02 Ma.

Our U–Pb age model is unable to accurately constraint the age of the lower boundary of the Kaiparowits Fm., as bentonites are notoriously absent from its lower unit, making the stratigraphic positions of our dated bentonites relative to the underlying Wahweap Fm. somewhat uncertain. However, a new U–Pb geochronologic study of the Wahweap Fm.^86^ has more reliably placed the Wahweap-Kaiparowits formation boundary at 77.29 ± 0.72 Ma, which is used here as the lowermost constraint for our age model (Fig. 4).

### San Juan Basin, New Mexico, USA

Only two of the collected bentonites from the Campanian of the San Juan Basin yielded zircons suitable for U–Pb analyses. These samples (SJB-1801 and SJB-1802, respectively) correspond to Ash J and Ash 2 of Fassett^107^ and both were collected from the Hunter Wash area, San Juan County, New Mexico. Ash 2 from the middle stratigraphic levels of the Fruitland Fm. yielded a weighted mean ^206^Pb/^238^U date of 75.166 ± 0.014 Ma, whereas Ash J from the uppermost Kirtland Fm. produced a weighted mean ^206^Pb/^238^U date of 73.496 ± 0.039 Ma (Table 1 and Fig. 3). While insufficient to construct a detailed age-stratigraphic model, these new ages bracket the main stratigraphic range of the Campanian vertebrate fauna in the San Juan Basin.

## Discussion

### High-resolution chronostratigraphy of the Campanian dinosaur record

The high-precision U–Pb zircon geochronology and Bayesian age models presented here provide high-resolution temporal frameworks for major Campanian vertebrate-bearing formations over a 1600-km latitudinal distance across the WIB. Our ash bed zircon ages significantly improve upon a substantial database of legacy ^40^Ar/^39^Ar ages from the WIB, which accumulated in a piece-meal fashion over nearly three decades (see Supplementary Table S1). A key advantage of the U–Pb CA-ID-TIMS method over alternative geochronologic techniques (e.g., ^40^Ar/^39^Ar and microbeam U–Pb dating) is that its measured isotopic ratios are metrologically traceable to the basic SI units of weight, time and radioactivity via a carefully calibrated isotopic tracer^128^^,^ which lacks the geologic (and age) complexities of natural mineral standards. Owing largely to the chemical abrasion technique^129^ and the use of EARTHTIME mixed U–Pb tracers^128,130^, modern CA-ID-TIMS geochronology is capable of generating highly reproducible ash bed ages with internal uncertainties as low as ± 12 ky in the Campanian (Table 1). The precision of individual zircon ^206^Pb/^238^U analyses (average of ± 72 kyr or 1.9‰ in this study) is crucial to the accuracy of the calculated weighted mean ages for bentonites, as it enables the detection of detrital and/or antecrystic zircon populations, which are present in many ash beds. The latter poses an important challenge to the application of U–Pb geochronology to the terrestrial stratigraphic record. An example of reproducibility of modern U–Pb CA-ID-TIMS ages between different laboratories is presented in the Methods. While ^40^Ar/^39^Ar geochronology continues to develop as a viable tool for high-resolution chronostratigraphy, the practice of repeatedly recalculating legacy ^40^Ar/^39^Ar ages (e.g., Fowler^123^) using new and evolving parameters only compounds embedded analytical issues (see discussion above) and is arguably counterproductive to building a reliable age framework.

Whereas individual vertebrate-bearing formations of the WIB may represent several million years of terrestrial deposition, the fossils are not uniformly distributed throughout each unit. For example, the megaherbivore dinosaur assemblages of the Dinosaur Park Fm. are thought to reflect turnovers with durations on the order of 300 kyr^45^. Each assemblage zone may in turn consist of multiple fossil horizons or bone-beds, which may have been separated from each other by only a few tens of thousands of years. It is therefore imperative for the employed geochronologic technique to have uncertainties compatible with the desired stratigraphic resolution. The level of age precision provided in this study is also necessary to explore possible links between faunal/floral turnovers, depositional sequences tied to sea level cyclicity and paleoenvironmental change driven by Milankovitch cycles.

The fossil-rich stratigraphic intervals of the Dinosaur Park, Two-Medicine, Judith River and Kaiparowits formations are known to coincide with an increase in the abundance of bentonites and/or volcanic components in the associated alluvial sediments^36,83,105^. Our geochronology indicates that this lithologic change occurred at ca. 76.4 Ma, broadly coincident with the Dinosaur Park Fm. in southern Alberta, Coal Ridge Member of the Judith River Fm. in Montana and the middle unit of the Kaiparowits Fm. in southern Utah. A likely explanation for the temporal association between volcanism and fossil abundance would be environmental change and enhanced habitability (ecological expansion), either as a direct result of elevated volcanic activity, or in association with foreland basin tectonic and landscape evolution. It has been hypothesized that extensive volcanism can impact atmospheric circulation, hydrological cycles, and nutrient transport in the environment, impacting the terrestrial biota^131^. Alternatively, diagenetic characteristics of volcanogenic sediments may favor vertebrate fossil preservation and increase their occurrence. While vertebrate fossil burial and preservation in pyroclastic flows are well documented^132^, the taphonomic influence of distal tuffaceous sediments on fossil preservation is less understood. If the latter plays the dominant role, the apparent stratigraphic ranges of fossil occurrences in the above formations may underestimate their true temporal ranges. More focused research is necessary to resolve the paleoecologic versus taphonomic influence of volcanism on vertebrate fossil occurrences.

**Figure 6:**
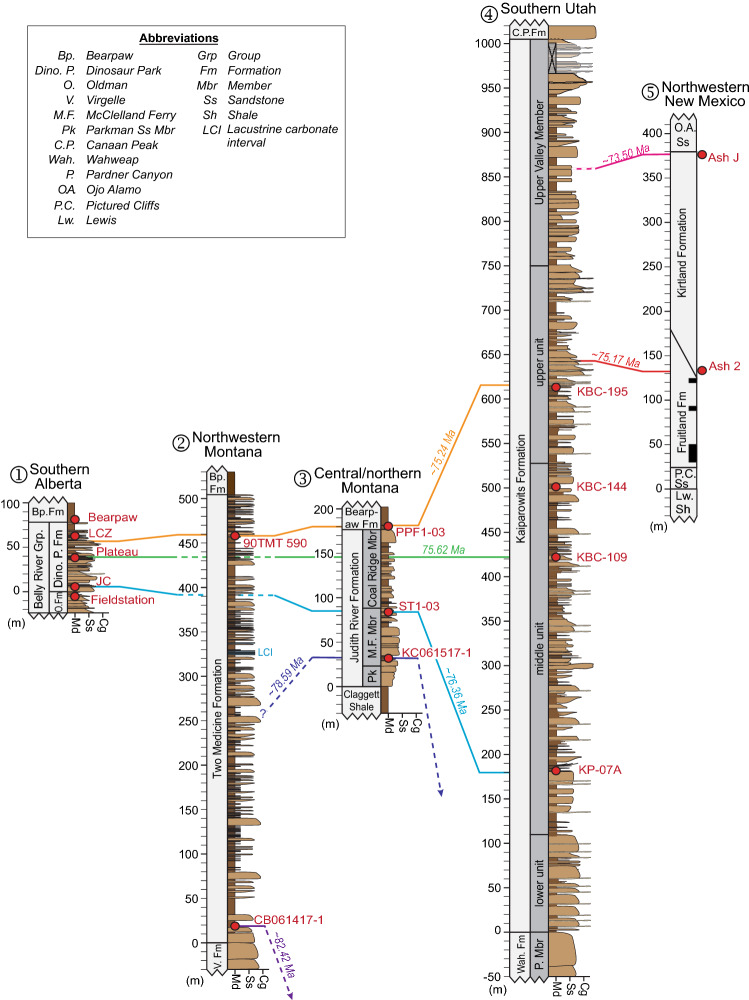
Temporal correlations among five principal Campanian fossil-bearing formations of the Western Interior Basin, based on the age models of this study. Stratigraphic columns are drawn to the same scale. Black is coal bed. Red dots mark ash beds with U–Pb ages connected by color-coded temporal correlation lines; dashed lines signify age projection in the absence of a quantitative age-stratigraphic model. Lithostratigraphy after: (1) Eberth and Hamblin^36^, (2) Rogers^50^, (3) Rogers et al.^83^, (4) Roberts et al.^105^, (5) Fassett and Heizler^114^, Sullivan and Lucas^144^.

Our new age models for the Campanian formations elucidate their detailed depositional histories and allow a quantitative assessment of associated rock accumulation rates, demonstrating that these rates were highly variable across the WIB during the Campanian. The average rock accumulation rates (disregarding post-depositional sediment compaction) vary dramatically among our studied formations, ranging from 36.6 ± 0.4 m/Myr for the Dinosaur Park Fm. to 372 ± 15 m/Myr in the Kaiparowits Fm. The rates for the Judith River and Two Medicine formations are 44.2 ± 0.5 m/Myr and 62.1 ± 0.7 m/Myr, respectively, indicating an anomalously high accumulation rate for the Kaiparowits Fm. (Fig. 6). Overall, our results reveal disparate basin evolution and subsidence histories throughout the WIB.

Drastically different rock accumulation rates across the WIB translates into potentially variable depositional settings, and possibly dissimilar foreland basin architectures. Nevertheless, the main vertebrate fossil-bearing intervals of the Kaiparowits (77.24–75.02 Ma), Judith River (76.32–75.22 Ma) and Dinosaur Park (76.61–75.04 Ma) formations largely overlap in their age ranges (Figs. 2 and 6). The same generally holds true for the Two Medicine Fm., although more work is needed to tie all of its fossil occurrences to the current age-stratigraphic model. Interestingly, this fossil-bearing interval occurs distinguishably later in the San Juan Basin (ca. 75.2–73.5 Ma). A lack of correlation between fossil abundance and depositional setting (e.g., burial rates) argues against a strong depositional control on fossil preservation and suggests that fossil distributions in the Campanian formations to the first order reflect paleoecologic richness (see discussion below).

### Concept of intracontinental dinosaur endemism and latitudinal provinciality

It has been hypothesized that coeval Late Cretaceous vertebrate fauna (and flora) throughout the Laramidia can be divided into distinct, latitudinally arrayed ‘provinces’^5,6^. Whereas the same major clades (e.g., hadrosaurids, ceratopsids, ankylosaurids, tyrannosaurids) are present in dinosaur assemblages throughout the WIB, the northern and southern assemblages are thought to be distinct at the genus and species levels. Sampson et al.^7,99^ argued that latitudinal provinciality can be best observed among the chasmosaurine and centrosaurine ceratopsids of the late Campanian of the Laramidia, in light of new taxa being discovered from the Kaiparowits Fm. in the south. The biogeographic analyses of Gates et al.^133^ based on multivariate analyses of terrestrial vertebrate taxa (including freshwater fish, reptiles/amphibians and mammals in addition to dinosaurs) added further support to a late Campanian latitudinal gradient. Rapidly growing datasets and expanded collections of the Laramidian fauna and flora^2,7,35,45,54–56,102,104,112,113,133–140^ provide new opportunities for analyzing paleobiogeographic patterns, including biotic endemism, with increasing resolution. It has been suggested that physiographic elements such as the Elkhorn Mountains volcanic field and their impact on the adjacent alluvial systems could have generated distinct Laramidian ecosystems capable of engendering faunal divergence^141^. The possible extent and duration of such ecological partitioning, however, have not been demonstrated. The alternative view is that the observed biogeographic zonation was driven by climatic gradients (e.g., mean annual temperature and rainfall) across the meridional WIB expanse^133,142^.

The notion of WIB vertebrate endemism has been challenged on the grounds of fossil sampling bias, ambiguous correlations among diachronous units, and arbitrary taxonomic classifications^123,143–145^. More specifically, Dean et al.^146^ suggested that grouping non-contemporaneous fossil-bearing geological units into large time bins in order to assess faunal richness and diversity on a regional scale can give the appearance of high endemicity to the Campanian dinosaurs of WIB. Similarly, Maidment et al.^147^ highlighted the current challenges associated with investigating large scale patterns of endemism for the same reasons, but highlighted the potential of smaller, focused investigation of specific groups within well calibrated stratigraphic intervals.

Rigorous examination of the Campanian dinosaur provinciality hypotheses requires a systematic, data-driven approach involving careful stratigraphic, temporal and statistical considerations, which have been all or in part absent from pervious paleobiogeographic reconstructions. We maintain that a critical evaluation of dinosaur faunal distribution and diversity will rely on the following variables:

1. *Environmental control on fossil preservation and accumulation* Changes in the depositional environment and landscape induced by tectonic and climatic drivers can not only influence the preservation potential of vertebrate fossils, but their taxonomic composition, as well^148^. For example, an apparent decline in dinosaur diversity prior to the end-Cretaceous extinction^149,150^ coincides with the end of the Cretaceous sea level mega cycle characterized by accelerated regression starting from the late Campanian^10^, suggesting a potential causal link^146^. At a local scale, different depositional settings can selectively preserve different aspects of faunal assemblages, leading to systematic variations in taphonomic composition as a function of fluvial facies^83,151^. These fossil-facies associations must be taken into account when investigating paleoecology, paleobiogeography and patterns of dinosaur distribution and turnover.
2. *Sampling bias* The intensity of fossil collection within and between formations is a source of potential bias when considering provinciality arguments. For instance, collecting of vertebrate fossils in the Dinosaur Park and Judith River formations began during the mid-late 1800s, whereas the first comprehensive collecting efforts in the Kaiparowits and Wahweap formations began nearly a century later. However, it can be difficult to assess the overall collecting effort/intensity among formations regardless of when collection began. Not only does this lead to potential cross-formation bias, but there are clearly biases in collecting efforts within individual formations, as well. For example, in a comprehensive biostratigraphic study of the lower Campanian Wahweap Fm. in southern Utah (Kaiparowits Plateau), Beveridge et al.^86^ noted that the majority of dinosaur localities were clustered around vehicle-accessible roads, which were built predominantly above steep cliff-forming sandstone horizons.
3. *High-resolution age control* Undoubtedly, imprecise (and inaccurate) age constraints can lead to oversimplified—if not erroneous—paleobiologic and paleoecologic reconstructions. Therefore, the focus of high-resolution radioisotopic geochronology must be fossil-rich stratigraphic intervals, and not merely the formations that host them. This approach is necessary to quantitatively identify gaps in the fossil record and accurately elucidate patterns of evolutionary change, albeit not always possible, due to scarcity of datable ash beds in some fossiliferous intervals.

The present contribution was aimed primarily at developing a robust, internally consistent temporal framework for fossiliferous strata across the WIB (item 3 above). Our results reveal a remarkable overlap in age among the main fossil-bearing intervals in the Kaiparowits, Judith River, Two Medicine, and Dinosaur Park formations, which predate the apparently younger fossil-rich interval in the Fruitland/Kirtland formations (Figs. 2 and 4). The strong temporal correlation across most of these key intervals refutes the argument of Fowler^123^ that the apparent latitudinal provinciality of the Campanian dinosaur fauna is simply an artefact of erroneous age interpretations. Moreover, we have demonstrated that the largely coeval fossil assemblages across the WIB are preserved in rocks deposited in different basinal settings with drastically dissimilar accumulation rates. Represented in these assemblages, however, are broadly similar dinosaur lineages (e.g., hadrosaurids, ceratopsids and tyrannosaurids), as well as a host of other vertebrate and invertebrate fossil groups. This observation contradicts a strong taphonomic control on taxonomic richness and diversity and suggests that the observed WIB-wide fossil associations may serve as good representatives of the true Campanian biogeography and paleoecology.

In the absence of compelling evidence for taphonomic bias (1 above), and with the improved chronostratigraphic framework presented here (3 above), limited sampling and collection bias (2 above) emerges as the chief source of uncertainty in resolving the Campanian paleobiogeography and paleoecology of dinosaur fauna. Collection bias can emanate from a combination of prospecting history, outcrop exposure and quality, land access, and fossil type preferences and can occur both within and between formations. Sustained efforts in systematic fossil collection integrated with high-resolution stratigraphy, sedimentology, taphonomy, and geochronology throughout the WIB promise to offer new insights into the evolutionary patterns of dinosaurs during the zenith of their diversity in the Campanian.

## Conclusions

New U–Pb CA-ID-TIMS geochronology based on 16 volcanic ash beds from several richly fossiliferous Upper Cretaceous units across North America’s Western Interior Basin is used to construct a high-resolution chronostratigraphic framework across 1600 km of Campanian terrestrial ecosystems. This significantly enhanced temporal context allows robust correlations among geographically distant fauna as a basis for addressing long standing questions regarding dinosaur paleogeography, paleoecology and evolution during the Campanian. Our results demonstrate a remarkable overlap in age among most of these Campanian fossil-producing intervals from Utah to Alberta, refuting inferences that the proposed latitudinal provinciality of dinosaur taxa is simply an artefact of age misinterpretation.

The contemporaneous occurrence of abundant and diverse fossil assemblages in a latitudinal array of depocenters characterized by a variety of depositional settings argues against a dominant taphonomic control on fossil preservation and lends support to the notion that the Campanian dinosaur assemblages are indeed credible representatives of paleoecologic richness and diversity. An improved and high-resolution temporal framework helps identify gaps in the fossil record and facilitate further targeted collecting with the goal of diminishing fossil sampling bias, which remains the chief source of uncertainty in understanding large-scale patterns of faunal evolution during the ‘zenith’ of dinosaur diversity.

## Methods

### Stratigraphy

Detailed **s**tratigraphic sections were measured in the context of previous studies^25,29,36,37,50,83,90,92,105,114^, which had identified and sampled a number of target bentonite beds. New bentonite samples aimed at U–Pb zircon geochronology were collected during the 2016 and 2017 field seasons and their locations where placed within the corresponding stratigraphic frameworks by using the global positioning system (GPS) and by measuring distance relative to known stratigraphic horizons (Supplementary Fig. S2). Sample selection strategy was based primarily on bracketing the stratigraphic intervals of interest, including lithostratigraphic contacts and sequence stratigraphic surfaces of significance, in addition to fossiliferous horizons.

### U–Pb geochronology

Samples of bentonite collected for zircon geochronology were on the order of 4 to 6 kg in weight and were carefully excavated from at least 2 cm above the bottom to avoid the basal crystal-lithic tuff horizon rich in reworked/detrital grains. Sampled bentonites were 5–90 cm-thick and were characteristically clay rich with variable silt- and sand-sized contents, and commonly formed recessed benches or hilltop flats in the local landscape. The typical bentonite is homogeneous, with a lustrous olive-green color on fresh surfaces and has no visible lamination or parting. Samples were processed in the lab by soaking in water for 48 h, followed by complete liquefaction in a blender and gradual clay disintegration and removal in a sonic dismembrator device^152^. Heavy-mineral concentrates were obtained using magnetic as well as high-density liquid separation. Final zircon selection was carried out by hand picking under a binocular microscope.

Bentonite samples contained to varying degrees mixed populations of zircon characterized by a variety of grain sizes and morphologies. These ranged from equant/sub-equant grains to prismatic and acicular zircon with high aspect ratios and up to 500 µm in length. Preference in zircons selection was given to sharply faceted, prismatic/acicular zircon that contained elongate glass (melt) inclusions parallel to their long axis (Supplementary Fig. S1). Past experience has demonstrated that these grains typically yield the youngest dates in samples characterized by mixed zircon populations and this has proven an efficient screening technique for xenocrystic and/or far transported detrital zircon grains^153^.

Zircons selected for U–Pb analysis were pre-treated by a chemical abrasion technique modified after Mattinson^129^, which involved thermal annealing in a 900 °C furnace for 60 h, followed by partial dissolution (leaching) in concentrated hydrofluoric acid (HF) in order to mitigate the effects of Pb-loss in zircon that often result in anomalously young dates. For leaching, annealed zircons were loaded with ca. 75 µl of 29 M HF into 200 µl FEP Teflon® microcapsules, placed within a high-pressure Parr® vessel and left in a 210 °C oven for 12–13 h. This is considered a rather aggressive leach schedule, but proven necessary as a remedy for persistent Pb loss in certain zircons^154^. The leached grains were transferred into 3 ml Savillex® FEP beakers and fluxed in successive steps in a dilute HNO_3_ solution and in 6 N HCl over a hot plate (1 h per step), with each step followed by agitation in an ultrasonic bath (1 h) and rinsing with several milliliters of ultra-pure water to remove the leachates. Thoroughly rinsed zircon grains were loaded back into their microcapsules, spiked with the EARTHTIME ET2535 mixed ^202^Pb-^205^Pb-^233^U-^235^U isotopic tracer^128,130^ and dissolved completely in 29 M HF at 210 °C for 48 h.

Dissolved Pb and U were chemically separated using a miniaturized HCl-based ion-exchange chemical procedure modified after Krogh^155^, using 50 µl columns of 1 × 8 anion-exchange resin. Purified Pb and U were loaded with a silica gel—H_3_PO_4_ emitter solution^156^ onto single, degassed Re filaments and their isotopic ratios were measured on the Isotopx X62 multi-collector thermal ionization mass spectrometer equipped with a Daly photomultiplier ion counting system at MIT. Pb isotopic measurements were made on monoatomic Pb ions in a peak-hopping mode on the ion counter, whereas U isotopes were measured as UO_2_^+^ in a static mode on three Faraday detectors simultaneously.

A total of 118 single zircons were analyzed from 16 bentonite beds from the four WIS study localities. Complete Pb and U isotopic data are given in Supplementary Table S2. Data reduction, as well as calculation of U–Pb dates and propagation of uncertainties were accomplished using the Tripoli and ET_Redux applications^157,158^. Measured isotopic ratios were corrected for mass-dependent isotope fractionation in the mass spectrometer using the tracer ^202^Pb/^205^Pb and ^233^U/^235^U isotopic ratios, as well as for U and Pb contributions from the spike and laboratory blanks. Common Pb in the analyses averaged 0.37 pg, all of which was attributed to laboratory blank, and its isotopic composition was determined from long-term measurements of the total procedural Pb blank in the lab (see Supplementary Table S2 footnotes). The radiogenic ^206^Pb concentrations were also corrected for initial ^230^Th disequilibrium in magma using a magma initial Th/U model ratio of 2.8 ± 1.0 (2σ). This range of Th/U ratios encompasses all likely compositions of the magma source of an intermediate to felsic tuff^159^. The Pb isotopic ratios were corrected for isobaric interferences from Tl and BaPO_2_ on mass 205 by monitoring masses 203 and 201, respectively, and using natural isotopic abundances of ^138^Ba and ^205^Tl. Measured U isotopic ratios were also corrected for isobaric interference of ^233^U^18^O^16^O with ^235^U^16^O^16^O using an ^18^O/^16^O ratio of 0.00205 ± 0.00004 (2σ), which has been determined from long-term measurements of 272/270 mass ratio from large U loads. The present-day natural U isotopic composition of 137.818 ± 0.044 (2σ) was used in data reduction following Hiess et al.^160^.

In general, ^206^Pb/^238^U dates are considered the most precise and accurate in high-precision U–Pb geochronology as they are independent of suspected inaccuracy of the ^235^U decay constant^161,162^, which potentially biases the ^207^Pb/^235^U or ^207^Pb/^206^Pb dates. Since the presence of xenocrystic/antecrystic or detrital (reworked) zircon in volcanic ash cannot be ruled out, our sample ages are derived from the weighted mean ^206^Pb/^238^U date of a statistically coherent cluster of the youngest zircon analyses in each sample, after excluding older analyses (outside 2σ analytical uncertainty) that are considered xenocrystic or detrital. With only three exceptions, the youngest cluster comprised between 58 and 100% of the total analyses from the sample (Table 1), rendering some calculated dates more statistically robust than others. Uncertainties in calculated ^206^Pb/^238^U dates are reported at 95% confidence level (Table 1 and Fig. 3) and in the ± *X*/*Y*/*Z* Ma format, where *X* is the internal (analytical) uncertainty in the absence of all external errors, *Y* incorporates *X* and the U–Pb tracer calibration errors and *Z* includes the latter as well as the decay constant errors of Jaffey et al.^163^. The external uncertainties must be taken into account if the results are to be compared with U–Pb dates obtained in other laboratories with different tracers, with different techniques (e.g., microbeam U–Pb), or ones derived from other isotopic chronometers (e.g., ^40^Ar/^39^Ar). However, for establishing a chronology based on the results of this study alone (or from other studies that used the same U–Pb isotopic tracer), only the analytical uncertainties (*X*) need to be considered.

The reproducibility of U–Pb CA-ID-TIMS geochronology among different laboratories is often tested using large grains (or fragments) of a well-characterized zircon standard^164^, but it is rarely demonstrated based on lithologically complex samples such as bentonites. The Bearpaw tuff (our sample IL082717-1) from the basal Bearpaw Fm. in the Dinosaur Provincial Park, Alberta, was independently sampled and analyzed in the Jack Satterly Geochronology Laboratory at the University of Toronto, using an EARTHTIME isotopic tracer and essentially similar analytical procedures, and yielded a weighted mean ^206^Pb/^238^U date of 74.308 ± 0.031/0.050/0.130 Ma^126^. The latter is older than the Bearpaw tuff age reported here (74.289 ± 0.014/0.024/0.083 Ma) by 0.26‰, well within analytical uncertainty. However, Eberth and Kamo^126^ chose a natural ^238^U/^235^U ratio of 137.88^118^ and a magma Th/U ratio of 4.2 in their U–Pb age calculations. If the same data reduction parameters are used in both studies, the offset between the two ages will be reduced to a nominal 0.16‰.

### Age-stratigraphic modelling

In order to construct robust chronostratigraphic frameworks for the fossil-bearing successions of the Belly River Group (Alberta), Judith River Fm. (Montana) and Kaiparowits Fm. (Utah), we employed a Bayesian age-stratigraphic model using the Bchron software package^165,166^. The model utilizes the weighted mean dates of all analyzed samples and their relative stratigraphic positions to extrapolate the age of any given stratigraphic horizon of interest. The underlying Markov chain Monte Carlo rejection algorithm of Bchron takes into account possible changes in the rock accumulation rate and results in more objective stratigraphic age uncertainties that the conventional linear extrapolation or spline-fit methods. The Bchron age model is shown with its median (solid) line and 95% confidence interval (shaded band) in Figs. 4 and 5. Code scripts, input data, and numerical model outputs are included in Supplementary Table S3.

### Repository

Mineral and zircon separates from the processing of tuff samples are archived at the MIT Isotope Lab in Cambridge, Ma.

## Supplementary Information

Supplementary Information 1.

Supplementary Information 2.

Supplementary Information 3.

Supplementary Information 4.

Supplementary Information 5.

## Data Availability

All data generated and analyzed as part of this study are included in this published article and its Supplementary Information files.
