# Supplementary material for: Calibrating the zenith of dinosaur diversity in the Campanian of the Western Interior Basin by CA-ID-TIMS U–Pb geochronology
Source: Sci Rep. 2022 Sep 26;12:16026. doi: 10.1038/s41598-022-19896-w (PMC9512893; doi:10.1038/s41598-022-19896-w)
Supplement: Supplementary file 1 — Supplementary Information 1. [file 41598_2022_19896_MOESM1_ESM.pdf]

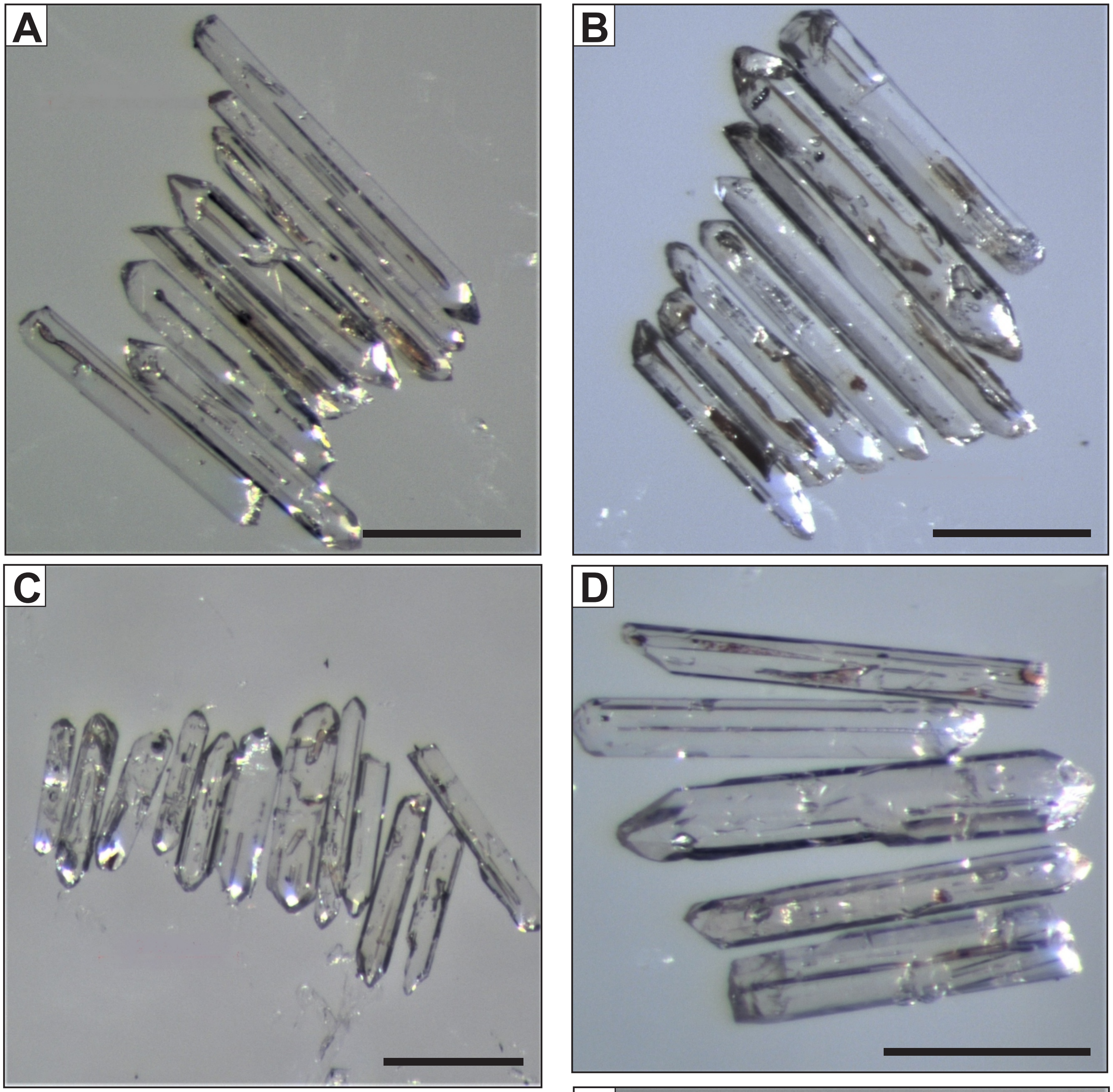

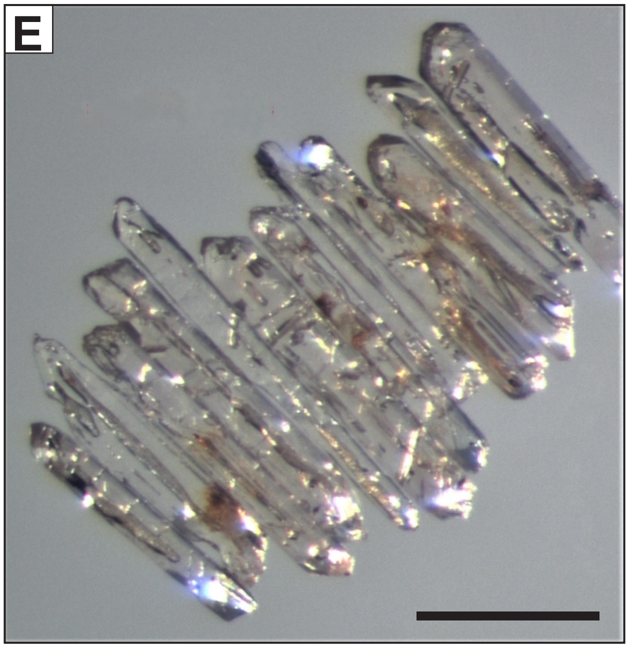

FIGURE S1: Microscope images of analyzed zircons from selected tuff beds of the Western Interior Basin: A) sample IL082717-1 (Bearpaw tuff) from Dinosaur Provincial Park, Alberta, Canada; B) sample 90TMT-590-U60 from Two Medicine Formation type area, northwestern Montana; C) sample KC061517-1 from Kennedy Coulee, northern Montana; D) sample KBC-144 from Kaiparowits Plateau, southern Utah; and D) sample SJB-1802 (Ash 2) from San Juan Basin, northwestern New Mexico. Scale bar is 200 µm. Note the abundance of glass (melt) inclusions parallel to the long axis of the selected zircon
