# Supplementary material for: Calibrating the zenith of dinosaur diversity in the Campanian of the Western Interior Basin by CA-ID-TIMS U–Pb geochronology
Source: Sci Rep. 2022 Sep 26;12:16026. doi: 10.1038/s41598-022-19896-w (PMC9512893; doi:10.1038/s41598-022-19896-w)
Supplement: Supplementary file 2 — Supplementary Information 2. [file 41598_2022_19896_MOESM2_ESM.pdf]

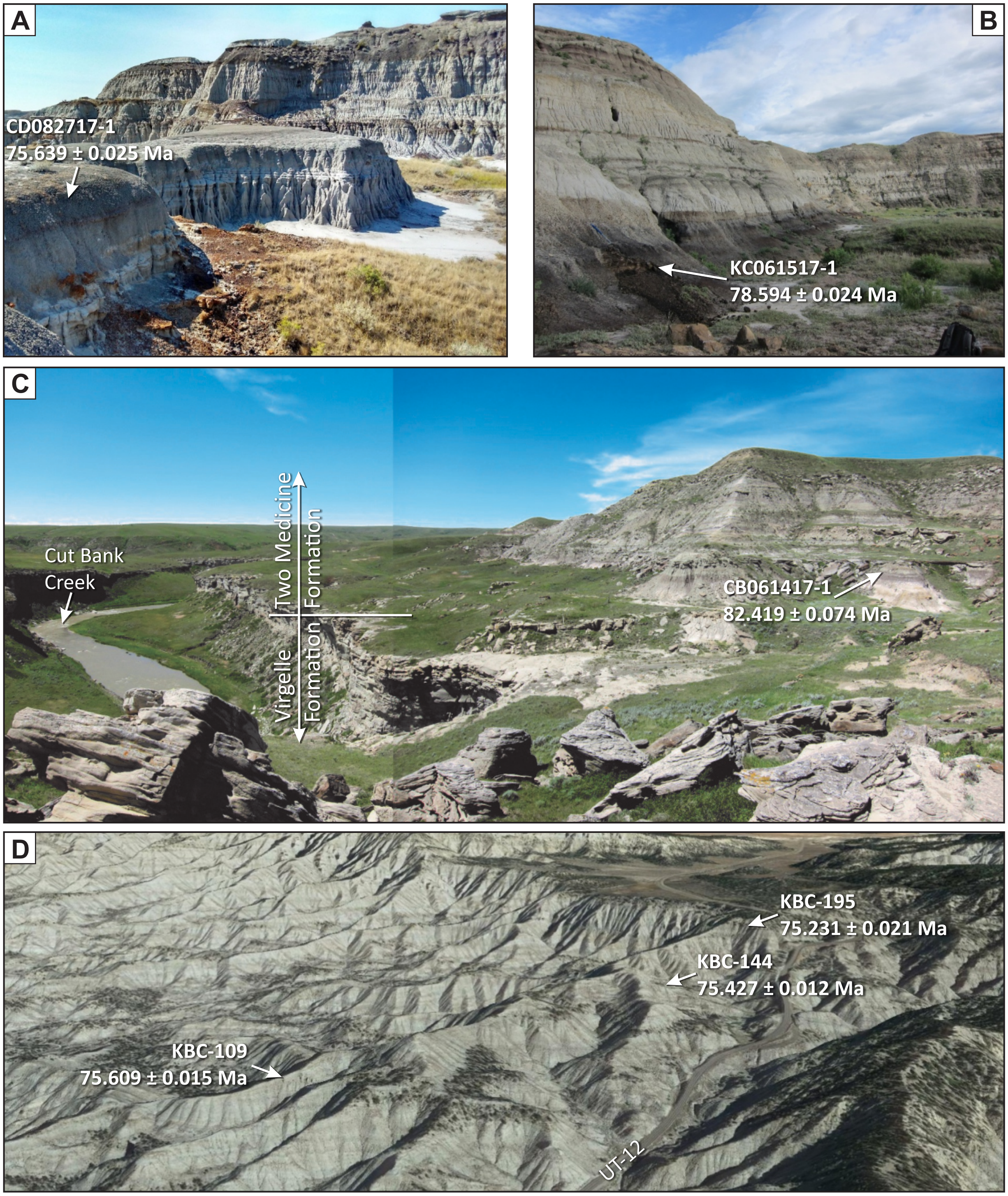

Figure S2. Outcrop views of Western Interior Basin formations investigated as part of this study, showing dated tuff locations: A) Fieldstation tuff of Dinosaur Park Formation at the Cathedral locality of Dinosaur Provincial Park, Alberta, Canada, B) tuff within Coal Marker A bed of Judith River Formation at Kennedy Coulee, northern Montana, and C) basal tuff of Two Medicine Formation at Cut Bank Creek, western Montana (photos taken by Jahandar Ramezani). D) Satellite image of the Kaiparowits Blues Creek locality in southern Utah showing tuffs in the middle and upper units of Kaiparowits Formation (Map Data: Google, ©2022 Maxar Technologies, USDA/FRPAC/GEO).
