# Supplementary material for: Calibrating the zenith of dinosaur diversity in the Campanian of the Western Interior Basin by CA-ID-TIMS U–Pb geochronology
Source: Sci Rep. 2022 Sep 26;12:16026. doi: 10.1038/s41598-022-19896-w (PMC9512893; doi:10.1038/s41598-022-19896-w)
Supplement: Supplementary file 3 — Supplementary Information 3. [file 41598_2022_19896_MOESM3_ESM.pdf]

TABLE S1. Compilation of previosuly published radioisotope geochronology from the upper Campanian ash beds of the Western Interior Basin.

Sample Name	Formation / Member	Locality		Elevation (m)	Reported Age (Ma)	Internal error ± (2σ)	Method (mineral)[1]	Decay constant	Mineral standard (age in Ma)	Acquisition	Reference	Laboratory[6]
		Latitude	Longitude									
Southern Alberta												
BpB	Bearpaw Fm.	50.755531°N	111.381729°W	-	74.308	0.031	U-Pb (zircon) CA-IDTIMS	Jaffey et al. (1971)	n/a	weighted mean date	Eberth & Kamo (2020)	JSGL
Onefour tuff upper	Dinosaur Park Fm.	49°09'13.43"N	110°24'35.30"W	150	74.9	0.2	Ar-Ar (sanidine)	Steiger & Jäger (1977)	MMhb-1 (520.4 Ma)[2]	total fusion	Eberth & Hamblin (1993)	BGC
DPP tuff upper	Dinosaur Park Fm.	50°49'43.25"N	111°19'24.03"W	102	75.2	0.6	Ar-Ar (sanidine)	Steiger & Jäger (1977)	MMhb-1 (520.4 Ma)[2]	total fusion	Eberth & Hamblin (1993)	BGC
Onefour tuff lower	Oldman Fm.	49°09'13.43"N	110°24'35.30"W	80	76.2	0.4	Ar-Ar (sanidine)	Steiger & Jäger (1977)	MMhb-1 (520.4 Ma)[2]	total fusion	Eberth & Hamblin (1993)	BGC
DPP tuff lower	Oldman Fm.	50°49'43.25"N	111°19'24.03"W	54	76.5	1.0	Ar-Ar (sanidine)	Steiger & Jäger (1977)	MMhb-1 (520.4 Ma)[2]	total fusion	Eberth & Hamblin (1993)	BGC
Plateau Tuff	Dinosaur Park Fm.	50°44'46.85"N	111°29'18.65"W	64	76.11	0.44	Ar-Ar (sanidine)	Steiger & Jäger (1977)	MMhb-1 (520.4 Ma)[2]	total fusion	Thomas et al. (1990)	BGC
Montana - East												
PPF1-03	Bearpaw Fm. (basal)	47°43'28.7"N	108°56'37.3"W	180	75.21	0.24	Ar-Ar (sanidine)	Min et al. (2000)	FCT (28.201 Ma)[5]	total fusion	Rogers et al. (2016)	BGC
ST1-03	Judith River Fm. (middle)	47°45'37.2"N	109°19'46.9"W	85	76.24	0.36	Ar-Ar (sanidine)	Min et al. (2000)	FCT (28.201 Ma)[5]	total fusion	Rogers et al. (2016)	BGC
WHB1-11	Judith River Fm. (middle)	47°44'57.9"N	108°56'42.6"W	70	76.17	0.14	Ar-Ar (sanidine)	Min et al. (2000)	FCT (28.201 Ma)[5]	total fusion	Rogers et al. (2016)	BGC
84MG8-3-4	Judith River Fm./McClelland Ferry Mbr.	S24, T37N	R8E	58	78.2	0.4	Ar-Ar (sanidine)	Steiger & Jäger (1977)	MMhb-1 (520.4 Ma)[2]	total fusion	Goodwin & Deino (1989)	BGC
84MG7-16-1	Judith River Fm./McClelland Ferry Mbr.	S19, T37N	R9E	26	78.5	0.4	Ar-Ar (sanidine)	Steiger & Jäger (1977)	MMhb-1 (520.4 Ma)[2]	total fusion	Goodwin & Deino (1989)	BGC
Montana - West												
Hardo Hill	Two Medicne Fm. (middle)	S15, T31N	R7W	265	77.52	0.38	Ar-Ar (sanidine)	Steiger & Jäger (1977)	FCT (28.02 Ma)[4]	total fusion	Foreman et al. (2008)	BGC
TM-6	Two Medicne Fm. (upper)	S8,T29N	R8W	500	74.08	0.19	Ar-Ar (plagioclase)	Steiger & Jäger (1977)	FCT (27.84 Ma)[3]	total fusion	Rogers et al. (1993)	BGC
TM-4	Two Medicne Fm. (upper)	S35, T32N	R8W	480	74.27	0.31	Ar-Ar (plagioclase)	Steiger & Jäger (1977)	FCT (27.84 Ma)[3]	total fusion	Rogers et al. (1993)	BGC
TM-4	Two Medicne Fm. (upper)	S35, T32N	R8W	480	74.1	1.4	Ar-Ar (sanidine)	Steiger & Jäger (1977)	FCT (27.84 Ma)[3]	total fusion	Rogers et al. (1993)	BGC
WOFS-U5	Two Medicne Fm. (lower)	S33, T32N	R6W	108	79.60	0.43	Ar-Ar (plagioclase)	Steiger & Jäger (1977)	FCT (27.84 Ma)[3]	total fusion	Rogers et al. (1993)	BGC
TM-3	Two Medicne Fm. (lower)	S28, T32N	R5W	105	79.77	0.55	Ar-Ar (plagioclase)	Steiger & Jäger (1977)	FCT (27.84 Ma)[3]	total fusion	Rogers et al. (1993)	BGC
RT/TM-7	Two Medicne Fm. (lower)	S28, T23N	R5W	105	80.00	0.56	Ar-Ar (plagioclase)	Steiger & Jäger (1977)	FCT (27.84 Ma)[3]	total fusion	Rogers et al. (1993)	BGC
03-NC-ABB	Two Medicne Fm. (upper)	19 km west of Choteau, MT		-	75.92	0.64	Ar-Ar (plagioclase)	Steiger & Jäger (1977)	FCT (28.03 Ma)[4]	step heating	Varricchio et al. (2010)	USGS
03-NC-ABB	Two Medicne Fm. (upper)	19 km west of Choteau, MT		-	74.0	1.8	U-Pb (zircon) SIMS	Jaffey et al. (1971)	R33 (418.9 ± 0.4 Ma)	youngest zircon	Varricchio et al. (2010)	USGS
multiple samples	Two Medicne Fm. (upper)	19 km west of Choteau, MT		-	75.9	1.4	U-Pb (zircon) SIMS	Jaffey et al. (1971)	R33 (418.9 ± 0.4 Ma)	youngest zircon	Varricchio et al. (2010)	USGS
Kaiparowits Plateau												
KBO-37	Kaiparowits Fm. (upper)	0424773$_{UTM}$	4167799 $_{UTM}$	790	74.21	0.36	Ar-Ar (sanidine)	Steiger & Jäger (1977)	FCT (28.02 Ma)[4]	total fusion	Roberts et al. (2005)	BGC
"	"	"	"	"	74.69	0.36	"	Min et al. (2000)	FCT (28.201 Ma)[5]	"	Roberts et al. (2013)	
KBC-144	Kaiparowits Fm. (middle)	0425382$_{UTM}$	4165351 $_{UTM}$	490	75.02	0.30	Ar-Ar (sanidine)	Steiger & Jäger (1977)	FCT (28.02 Ma)[4]	total fusion	Roberts et al. (2005)	BGC
"	"	"	"	"	75.51	0.30	"	Min et al. (2000)	FCT (28.201 Ma)[5]	"	Roberts et al. (2013)	
KBC-109	Kaiparowits Fm. (middle)	0424535$_{UTM}$	4165074 $_{UTM}$	420	75.02	0.30	Ar-Ar (sanidine)	Steiger & Jäger (1977)	FCT (28.02 Ma)[4]	total fusion	Roberts et al. (2005)	BGC
"	"	"	"	"	75.51	0.30	"	Min et al. (2000)	FCT (28.201 Ma)[5]	"	Roberts et al. (2013)	
KDR-5	Kaiparowits Fm. (lower)	0434999$_{UTM}$	4156941 $_{UTM}$	80	75.96	0.28	Ar-Ar (sanidine)	Steiger & Jäger (1977)	FCT (28.02 Ma)[4]	total fusion	Roberts et al. (2005)	BGC
"	"	"	"	"	76.46	0.28	"	Min et al. (2000)	FCT (28.201 Ma)[5]	"	Roberts et al. (2013)	
KP-07	Kaiparowits Fm. (middle)	37°26'10.88"N	111°41'46.94"W	190	75.97	0.36	Ar-Ar (sanidine)	Min et al. (2000)	FCT (28.201 Ma)[5]	total fusion	Roberts et al. (2013)	BGC
KP-07	Kaiparowits Fm. (middle)	37°26'10.88"N	111°41'46.94"W	190	76.264	0.046	U-Pb (zircon) CA-IDTIMS	Jaffey et al. (1971)	n/a	weighted mean date	Roberts et al. (2013)	MIT
KK0407	Kaiparowits Fm. (upper)	-	-	765	74.6	7.4	U-Pb (zircon) LA-ICPMS	Jaffey et al. (1971)	Sri Lanka (563.5 Ma)	weighted mean date	Lawton & Bradford (2011)	ALC
04JL05	Kaiparowits Fm. (upper)	-	-	625	75.6	1.8	U-Pb (zircon) LA-ICPMS	Jaffey et al. (1971)	Sri Lanka (563.5 Ma)	weighted mean date	Lawton & Bradford (2011)	ALC
KK0507	Kaiparowits Fm. (upper)	-	-	590	76.1	0.8	U-Pb (zircon) LA-ICPMS	Jaffey et al. (1971)	Sri Lanka (563.5 Ma)	weighted mean date	Lawton & Bradford (2011)	ALC
KK0607	Kaiparowits Fm. (middle)	-	-	415	76.8	0.9	U-Pb (zircon) LA-ICPMS	Jaffey et al. (1971)	Sri Lanka (563.5 Ma)	weighted mean date	Lawton & Bradford (2011)	ALC
KK0207	Kaiparowits Fm. (middle)	-	-	265	73.8	1.4	U-Pb (zircon) LA-ICPMS	Jaffey et al. (1971)	Sri Lanka (563.5 Ma)	weighted mean date	Lawton & Bradford (2011)	ALC
KK0107	Kaiparowits Fm. (lower)	-	-	65	79.2	1.7	U-Pb (zircon) LA-ICPMS	Jaffey et al. (1971)	Sri Lanka (563.5 Ma)	weighted mean date	Lawton & Bradford (2011)	ALC
San Juan Basin												
J	Kirtland / Fruitland Fm.	36.36446°N	108.13205°W	384	73.04	0.25	Ar-Ar (sanidine)	Steiger & Jäger (1977)	FCT (28.02 Ma)[4]	total fusion	Fassett (2000)	USGS
"	"	"	"	"	73.52	0.25	"	Min et al. (2000)	FCT (28.201 Ma)[5]	"	Fassett & Heizler (2017)	
J	Kirtland / Fruitland Fm.	36.36446°N	108.13205°W	384	73.34	0.12	Ar-Ar (sanidine)	Min et al. (2000)	FCT (28.201 Ma)[5]	step heating	Fassett & Heizler (2017)	NMGRL
H	Kirtland / Fruitland Fm.	36.36066°N	108.13822°W	359	73.37	0.18	Ar-Ar (sanidine)	Steiger & Jäger (1977)	FCT (28.02 Ma)[4]	total fusion	Fassett (2000)	USGS
"	"	"	"	"	73.85	0.18	"	Min et al. (2000)	FCT (28.201 Ma)[5]	"	Fassett & Heizler (2017)	
CR	Kirtland / Fruitland Fm.	37.19032°N	107.30849°W	277	74.25	0.13	Ar-Ar (sanidine)	Steiger & Jäger (1977)	FCT (28.02 Ma)[4]	total fusion	Fassett (2000)	USGS
"	"	"	"	"	74.74	0.13	"	Min et al. (2000)	FCT (28.201 Ma)[5]	"	Fassett & Heizler (2017)	
LP	Kirtland / Fruitland Fm.	36.98620°N	108.17851°W	238	74.3	0.38	Ar-Ar (sanidine)	Steiger & Jäger (1977)	FCT (28.02 Ma)[4]	total fusion	Fassett (2000)	USGS
"	"	"	"	"	74.79	0.38	"	Min et al. (2000)	FCT (28.201 Ma)[5]	"	Fassett & Heizler (2017)	
4	Kirtland / Fruitland Fm.	36.30969°N	108.18536°W	180	74.55	0.29	Ar-Ar (sanidine)	Steiger & Jäger (1977)	FCT (28.02 Ma)[4]	total fusion	Fassett (2000)	USGS
"	"	"	"	"	75.04	0.29	"	Min et al. (2000)	FCT (28.201 Ma)[5]	"	Fassett & Heizler (2017)	
2	Kirtland / Fruitland Fm.	36.29403°N	108.23017°W	136	74.56	0.13	Ar-Ar (sanidine)	Steiger & Jäger (1977)	FCT (28.02 Ma)[4]	total fusion	Fassett (2000)	USGS

"	"	"	"	"	75.05	0.13	"	Min et al. (2000)	FCT (28.201 Ma)[5]	"	Fassett & Heizler (2017)	
2	Kirtland / Fruitland Fm.	36.29403°N	108.23017°W	136	75.02	0.04	Ar-Ar (sanidine)	Min et al. (2000)	FCT (28.201 Ma)[5]	step heating	Fassett & Heizler (2017)	NMGRL
DEP	Kirtland / Fruitland Fm.	36.18938°N	108.1721°W	29	75.56	0.41	Ar-Ar (sanidine)	Steiger & Jäger (1977)	FCT (28.02 Ma)[4]	total fusion	Fassett (2000)	USGS
"	"	"	"	"	76.06	0.41	"	Min et al. (2000)	FCT (28.201 Ma)[5]	"	Fassett & Heizler (2017)	
DEP	Kirtland / Fruitland Fm.	36.18938°N	108.1721°W	29	76.14	0.12	Ar-Ar (sanidine)	Min et al. (2000)	FCT (28.201 Ma)[5]	total fusion	Fassett & Heizler (2017)	NMGRL
Huerfantino	Kirtland / Fruitland Fm.	36.23855°N	106.91627°W	0	75.76	0.34	Ar-Ar (sanidine)	Steiger & Jäger (1977)	FCT (28.02 Ma)[4]	total fusion	Fassett (2000)	USGS
"	"	"	"	"	76.26	0.34		Min et al. (2000)	FCT (28.201 Ma)[5]	"	Fassett & Heizler (2017)	

NOTES

[1]Where multiple minerals were analyzed from the same sample by the same chronometer, only the preferred mineral (e.g., sanidine) date is listed here.

[2]McClure Mountain syenite hornblende: Samson and Alexander (1987). Revised age for MMhb-1 is presently 523.98 ± 0.12 Ma (Schoene and Bowring, 2006).

[3]Fish Canyon Tuff sanidine: Deino and Potts (1990)

[4]Fish Canyon Tuff sanidine: Renne et al. (1998)

[5]Fish Canyon Tuff sanidine: Kuiper et al. (2008). Revised age for FCT is presently 28.176 ± 0.023 Ma (Phillips et al., 2022).

[6]ALC——Arizona Laserchron Center, BGC—Berkeley Geochronology Center, JSGL——Jack Satterly Geochronology Laboratory, NMGRL—New Mexcio Geochronology Research Laboratory, USGS—United States Geological Survey.

Steiger & Jäger (1977): λ^{40} = 5.543 E-10 yr^{-1}, λ_β = 4.962 E-10 yr^{-1}

Min et al. (2000): λ^{40} = 5.463 E-10 yr^{-1}, λ_β = 5.37 E-10 yr^{-1}
